# The Connection and Development of Unpredictability and Sensitivity in Maternal Care Across Early Childhood

**DOI:** 10.3389/fpsyg.2022.803047

**Published:** 2022-03-07

**Authors:** Eeva Holmberg, Eeva-Leena Kataja, Elysia Poggi Davis, Marjukka Pajulo, Saara Nolvi, Hetti Hakanen, Linnea Karlsson, Hasse Karlsson, Riikka Korja

**Affiliations:** ^1^FinnBrain Birth Cohort Study, Turku Brain and Mind Center, Department of Clinical Medicine, University of Turku, Turku, Finland; ^2^Department of Psychology and Speech-Language Pathology, University of Turku, Turku, Finland; ^3^Department of Psychology, University of Denver, Denver, CO, United States; ^4^Department of Pediatrics, University of California, Irvine, Irvine, CA, United States; ^5^Department of Child Psychiatry, University of Turku, Turku, Finland; ^6^Department of Psychology and Speech-Language Pathology, Turku Institute for Advanced Studies, University of Turku, Turku, Finland; ^7^Centre for Population Health Research, Turku University Hospital, University of Turku, Turku, Finland; ^8^Department of Psychiatry, University of Turku and Hospital District of Southwest Finland, Turku, Finland

**Keywords:** unpredictability, sensitivity, stability, depressive symptoms, anxiety symptoms

## Abstract

Both patterns of maternal sensory signals and sensitive care have shown to be crucial elements shaping child development. However, research concerning these aspects of maternal care has focused mainly on maternal sensitivity with fewer studies evaluating the impact of patterns of maternal behaviors and changes in these indices across infancy and childhood. The aims of this study were to explore how maternal unpredictability of sensory signals and sensitivity develop and associate with each other from infancy to toddlerhood and whether elevated maternal depressive and anxiety symptoms relate to maternal unpredictable signals and sensitivity in toddlerhood. The study population consisted of 356 mother–child dyads assessed at 30 months; a subset of 103 mother–child dyads additionally participated in 8 months assessment. Maternal unpredictability and sensitivity were assessed from video-recorded free-play episodes at 8 and 30 months. Maternal depressive and anxiety symptoms were assessed with questionnaires at gestational weeks 14, 24, 34 and 3, 6, 12, and 24 months. Mean level of mothers’ unpredictability decreased on average whereas sensitivity did not change between infancy and toddlerhood. Both maternal unpredictability and sensitivity showed moderate level of individual stability from infancy to toddlerhood and these two measures were modestly correlated within each age. Elevated maternal depressive and anxiety symptoms were not related to unpredictability but related to lower maternal sensitivity in toddlerhood. These results identify unpredictable sensory signals as a characteristic of parental care that is independent of standard quality measures and suggest that it may be less influenced by maternal depressive and anxiety symptoms.

## Introduction

Parental care is a critical aspect of early experiences that has long lasting consequences for child development. Decades of research, based on the foundational work of Bowlby (1969), has demonstrated the importance of sensitive care for cognitive and emotional function (Bowlby, 1969; Ainsworth et al., 1978; Masur et al., 2005; Sroufe, 2005; Easterbrooks et al., 2012; Biringen et al., 2014). Recent research has demonstrated that patterns of maternally derived sensory signals is potentially an additional component of caregiving behavior that shapes development (Davis et al., 2017, 2019; Noroña-zhou et al., 2020; Granger et al., 2021). However, research area is new, and more basic methodological research is needed to explore normative developmental pathway of maternal unpredictable sensory signals from infancy onward and its relations to traditional measurements of parenting quality. In the current study, we compare two distinct aspects of maternal care: unpredictability of maternal sensory signals (a more novel measure of parental care) and maternal sensitivity (a more traditional measure used in previous studies). More specifically, we compare how these aspects of maternal care develop from infancy to toddlerhood at group and individual levels. Moreover, we study whether maternal psychological distress, as one of the most studied risk factor for maternal caregiving quality in infancy, contributes to this development.

The novel study paradigm investigating the unpredictability of maternal sensory signals during interactions with her infant is parallel to experimental rodent research illustrating that unpredictable sensory signals from the dam to the pup cause alterations to neural circuits related to cognitive and emotional development of the pup (Brunson et al., 2005; Ivy et al., 2008; Rice et al., 2008; Molet et al., 2016). This has been tested in simulated poverty experiments, where restriction of nesting materials for rat dams was found to lead to fragmented and unsystematic caregiving behavior of the rat dams. In turn, unpredictable sensory signals from the dam to the pup were related to impaired memory functions and anhedonia in pups (Brunson et al., 2005; Ivy et al., 2008; Rice et al., 2008; Molet et al., 2016). Research has shown that visual, auditory and somatosensory brain circuits require patterns of modality-specific signals during sensitive periods in order to develop (Khazipov et al., 2004; Espinosa and Stryker, 2012; Takesian et al., 2018). For example, patterns of light are critical for normative development of the visual circuits (Espinosa and Stryker, 2012). Given the impact of patterns of sensory signals on the development of sensory circuits it is plausible that maternal sensory signals would be related to the development of child’s emotional and cognitive systems as well.

Recently, this novel parameter of maternal care, patterns, was applied to human development (Davis et al., 2017, 2019; Vegetabile et al., 2019). We applied an analytic method from information theory, entropy rate, where unpredictable sequences of maternal sensory signals (auditory, tactile, and visual) to the infant are characterized by high entropy rate. Specifically, maternal sensory signals while interacting with her infant are assessed and entropy rate is computed to characterize the predictability of the transitions between different maternal sensory signals throughout interaction episode with a child.

Rather than analyzing the quality of these signals (e.g., supportive vs. unsupportive signal) only patterns of these signals are calculated. In this regard, highly predictable mother would repeat a similar pattern of transitions between the signals throughout an interaction episode (e.g., mother speaks to the child, then touches the child and then shows a toy to the child and repeats this order of behaviors). In contrast, unpredictable mother would shift between signals (auditory, tactile, visual) randomly. A strength of this approach is that entropy rate to characterize unpredictability can be applied across species (Davis et al., 2019).

The theoretical basis of focusing on patterns of sensory signals derives from our understanding of the biological principle that patterned signals are critical for development of sensory circuits (Baram et al., 2012). Patterned sensory information is assessed on a timescale consistent with shaping neuronal activity and further in experimental systems, environmental signals modulate circuit formation and refinement via activity dependent engagement of synapses (Woo et al., 1997; Paolicelli et al., 2011; Schafer et al., 2012). Building on these biological principles, human research has shown that the unpredictable patterns of maternal sensory signals are associated with poorer child cognition and effortful control development (Davis et al., 2017, 2019) and with infant’s blunted cortisol response (Noroña-zhou et al., 2020). Interestingly, it was found that unpredictability contributed to child cognitive development beyond maternal sensitivity and it has been suggested that maternal sensitivity and the unpredictability of maternal sensory signals are moderately related, but separate aspects of parenting (Davis et al., 2017).

Maternal sensitivity has theoretical background in attachment theory and focuses on the emotional relations between a mother and a child. Maternal sensitivity refers mother’s ability to attune to infant’s non-verbal communication and regulate one’s own as well as child’s affective states (Ainsworth et al., 1978; Stern et al., 1985; Feldman, 2015). Moreover, sensitivity requires ability to recognize the child’s interaction cues, and to respond to them appropriately and timely enough from the child perspective. One widely used approach to explore maternal sensitivity in the mother–child interaction from infancy onward is emotional availability (EA) (Biringen, 2008; Biringen et al., 2014; Saunders et al., 2015), which is based on attachment theory (Bowlby, 1969; Ainsworth et al., 1978) and emotional theories (Emde, 1980). According to attachment theory, child secure attachment is achieved by an accessible and sensitive caregiving whereas insensitive, intrusive and hostile parenting may lead to insecure attachment pattern (Bowlby, 1969; Ainsworth et al., 1978). In turn, safe attachment pattern enables child to regulate emotions in the stressful situations (Easterbrooks et al., 2012; Biringen et al., 2014). Further, a large body of empirical research has shown that high maternal sensitivity is associated with cognitive and emotional development of the child (Easterbrooks et al., 2012; Biringen et al., 2014; Dunkel and Woodley Of Menie, 2019).

Research with maternal sensitivity suggests that consistent environment is crucial for child healthy development. In attachment theory, consistently available and responsive parenting is thought to facilitate secure attachment whereas unavailability of the caregiver or inconsistency in expressions of sensitivity associates with insecure attachment pattern in the child (Bowlby, 1969; Ainsworth et al., 1978; Biringen et al., 2014). Such research has focused on the consistency of the caregiver’s responses to infant signals. In comparison, unpredictability paradigm explores patterns of early parental signals at a microlevel, i.e., the predictability of the sensory signaling input from the mother to the child. We propose that patterns of maternal sensory information, at a moment-to-moment timescale is an additional process that shapes the development of neural circuits with implications for cognitive and emotional development.

Parent–child interaction changes when child transits from infancy to toddlerhood and child acquires motor, cognitive and language skills and starts to seek autonomy (Edwards and Liu, 2002). However, development of caregiving behaviors across child development is infrequently studied and there are no longitudinal studies exploring the normative development of unpredictable maternal sensory signals from infancy onward. There is some evidence showing that child developmental changes in toddlerhood may pose challenges for parenting behaviors that are reflected in the decreased maternal sensitivity on average in toddlerhood compared to infancy (Bornstein et al., 2011). However, also opposite results showing an increase in the maternal sensitivity on average between infancy and toddlerhood (Kemppinen et al., 2006) have been reported. Furthermore, previous research has also revealed moderate individual stability of maternal sensitivity throughout infancy and toddlerhood (Dallaire and Weinraub, 2005; Kemppinen et al., 2006; Bornstein et al., 2011; Hall et al., 2015). In a study of 893 mother–child dyads, the quality of mother-child interaction was evaluated annually from infancy until the child age of 6 years (Dallaire and Weinraub, 2005). In this study, maternal sensitivity showed moderate stability from infancy through childhood, whereas negative parenting did not, suggesting that positive caregiving behaviors may show more stability compared to negative aspects of caregiving (Dallaire and Weinraub, 2005).

Further, to better understand the development of maternal caregiving behaviors from infancy onward, it is crucial to explore maternal factors that may affect maternal behaviors during infancy and childhood. Chronically high maternal depressive and anxiety symptoms starting during pregnancy constitute a well-known risk factor for the quality of maternal caregiving behavior in infancy (Field, 2010, 2017, 2018; Stein et al., 2014), but longitudinal associations through toddlerhood and contributions of maternal mental health to unpredictability aspect of caregiving behavior are less studied. Depressive symptoms relate to lower maternal sensitivity and a higher amount of negative behavior during interactions with an infant (Easterbrooks et al., 2000; Lovejoy et al., 2000) whereas anxiety has been related to higher parental controlling behavior (Stein et al., 2012). In our own study from the present sample, prenatal anxiety was found to relate to higher maternal intrusiveness and postnatal depressive symptoms to lower maternal structuring in infancy, supporting previous findings (Hakanen et al., 2019). Only a few human studies have explored maternal mental health in relation with the unpredictability of maternal sensory signals. Concurrent maternal postnatal depressive and anxiety symptoms have been found to be associated with higher unpredictability in maternal sensory signals during infancy (Davis et al., 2017, 2019). Moreover, pre- and postnatal maternal anxiety symptoms, but not depressive symptoms, in combination with low maternal self-regulation capacity, have shown to be related to unpredictable sensory signals in maternal caregiving during infancy (Holmberg et al., 2020).

The main focus of this study was to explore the stability and relations of two different aspects of maternal caregiving behavior: maternal unpredictable sensory signals and maternal sensitivity, both during infancy and early childhood. Another aim was to study the contributions of maternal mental health to maternal behaviors.

Our research questions were:

(1) Do maternal unpredictability of sensory signals and sensitivity change between infancy (8 months) and toddlerhood (30 months) at group mean and individual level? Based on prior literature it is not clear whether overall levels of maternal behavior might change at the group level as the child develops (Kemppinen et al., 2006; Bornstein et al., 2011; Célia et al., 2018). However, we do predict maternal behavior during infancy would be moderately correlated with behaviors during toddlerhood (Dallaire and Weinraub, 2005; Kemppinen et al., 2006; Bornstein et al., 2011; Hall et al., 2015).
(2) How do unpredictability of maternal sensory signals and maternal sensitivity relate to each other during infancy (8 months) and toddlerhood (30 months)? Based on previous research it is hypothesized that maternal unpredictable signals and sensitivity are distinct aspects of maternal care but modestly associated (Davis et al., 2017).
(3) How do elevated maternal depressive and anxiety symptoms throughout pre- and postnatal periods (from gestational week 14 to 2 years postpartum) relate to maternal unpredictable sensory signals and sensitivity in toddlerhood (30 months)? We predict that elevated depressive and anxiety symptoms throughout pre- and postnatal period would relate to higher unpredictability and lower maternal sensitivity in toddlerhood (Easterbrooks et al., 2000; Lovejoy et al., 2000; Stein et al., 2012; Tietz et al., 2014; Davis et al., 2017, 2019).

## Materials and Methods

### Study Design and Participants

The sample was drawn from families participating in the FinnBrain Birth Cohort Study (Karlsson et al., 2018), where the main aim is to study the effects of early life stress on infant development. The study population (*n* = 3838 families) initially comprised of pregnant women (and their partners) recruited by a research nurse during the first trimester ultrasound (gestational week [GW] 12) between December 2011 and April 2015, by a research nurse (*n* = 3838 families).

It is usually considered that by 8 months of age the infant has formed attachment in a way that they differentiate between primary caretaker and other people and self-regulation capacity and executive functioning are actively forming. For toddler development, the typical measurement points are 2–2.5 years. It is one of the phases where many of the key developmental milestones such language, gross-motor skills and social skills are reached and measurable. The current study is a part of a larger The FinnBrain Birth Cohort Study follow-up that includes several assessments and study visits for families especially during the first year of life. Thus, for practical reasons, the measurement points and intervals needed to be balanced to avoid any extra strain for the families (e.g., 6 versus 8 months of age, of which both are commonly in use in infant studies assessing parent–child interaction and child development). Time points for cohort assessments have been selected partially in keeping with some other cohort studies to have comparable child developmental data from different cohorts. For all these reasons, for the early childhood neuropsychological assessments, study visits during infancy (8 months) and toddlerhood (30 months) were chosen.

A nested case-control population, i.e., a Focus Cohort was drawn from the main cohort and comprised mothers who self-reported high levels of prenatal anxiety and/or depressive symptoms (highest 25th percentile/“cases”) versus lower levels of prenatal anxiety and depressive symptoms (lowest 25th percentile/controls) and their infants. The Focus Cohort was established to study the effects of prenatal stress and thus to ensure sufficient variation in the symptom scores. The total sum score for the cut-off points for “cases” and “controls” were as follows: ≥12 and ≤6 for the Edinburgh Postnatal Depression Scale (EPDS) (Cox et al., 1987), ≥10 and ≤4 for the Symptom Checklist-90 (SCL-90) (Derogatis et al., 1973) anxiety subscale, and ≥34 and ≤25 points for Pregnancy Related Anxiety Questionnaire Revised 2 (PRAQ-R2) (Huizink et al., 2015). The criteria for being classified as a case were: scoring at least once above the selected threshold on two different questionnaires; or scoring at least twice above the selected threshold on the same instrument during pregnancy.

Focus Cohort families enriched by other actively participating cohort families were invited for Child Development and Parental Functioning Lab study visits when the child was 8 and 30 months of age. Study visits included observational assessment of mother-child interaction. At 8 months study visit, observation of mother–child interaction was added later leading to a smaller sample.

For the 8 months study visit 354 families were invited for the mother–child interaction assessment and 195 of them participated (55.1%). Non-participating mothers had a lower education level [χ^2^(2) = 14.07, *p* = 0.001] and were younger [*t*(313) = 2.35, *p* = 0.020] compared to the participating mothers. For the 30 months study visit 1042 families were invited and 415 of them participated (39.8%) for the interaction assessment. Non-participating mothers were less educated [χ^2^(2) = 23.38, *p* = 0.000], had lower monthly income [*t*(995) = 3.576, *p* < 0.000] and were younger [*t*(1040) = 4.154, *p* = 0.000] compared to participating mothers.

The mother–child interaction sample, with an available data from both maternal unpredictability and sensitivity assessments included 377 mother-child dyads at 30-month timepoint. At 30 months 38 assessments were excluded due to the following reasons: inadequate videotape, having participating fathers instead of mothers and two outliers in the unpredictability data. Moreover, at the 30-month timepoint, 21 participants were excluded due to missing data on the background information of the socioeconomic situation and child health (i.e., education, income, birthweight, Apgar scores). Of these participants, 103 had data available at 8 months of age.

Hence, the final interaction sample at 30 months included 356 mother–child dyads. Data gained from both measurement points included 103 mother–child dyads. Sample demographics are presented in Table 1.

**TABLE 1:** Demographic characteristics of the sample.

	Study participants *n* = 356	Subset with 8 months interaction data *n* = 103
**Education *n* (%)**		
High school	25.8	22.3
Polytechnics	31.2	35.9
University	43	41.7
**Monthly income *n* (%)**		
1500 or less	32.9	33
1501–2500	53.4	55.3
2501–3500	12.1	10.7
>3500	1.7	1.0
Primiparous *n* (%)	51.4	63.1
Maternal age at delivery *M* (*SD*)	31.04 (4.51)	31.06 (4.02)
Gestational weeks at birth *M* (*SD*)	39.87 (1.47)	39.75 (1.50)
Apgar 1 min *M* (*SD*)	8.71 (1.18)	8.66 (1.36)
Apgar 5 min *M* (*SD*)	9.04 (0.81)	9.01 (0.93)
Birth weight *M* (*SD*)	3602.43 (496.17)	3590.79 (467.32)
Infant sex (boy) (%)	56.2	51.5

### Procedure

Mothers filled out a background information questionnaire at GW 14, including education level and monthly income. Information on maternal age and perinatal status (gestational weeks at birth, Apgar scores, birth weight and infant biological sex at birth) was gained from the national birth registries (Data from national birth registries, National Institute for Health and Welfare^1^). Depressive symptoms questionnaires were filled out at GW 14, GW 24, and GW 34 and 3, 6, 12, and 24 months postpartum and anxiety questionnaires at GW 14, GW 24, and GW 34 and 3, 6, and 24 months postpartum. Mother–child interaction was assessed in a video-recorded free-play situation when the child was 8 and 30 months of age. At the 8 months study visit the mother was given a standard set of toys and asked to play 20 min together with her child as they would play at home. At 30 months study visit the play episode included a 10-min free-play and 5-min snack time. The video-recorded free-play situations at both timepoints were analyzed using two different methods/coding strategies (see Measures below). The Ethics Committee of the Hospital District of Southwest Finland approved the study protocol.

### Measures

#### Unpredictability of Maternal Sensory Signals (i.e., Entropy Rate)

Maternal sensory signals (i.e., auditory, tactile, visual) were coded from the 10-min free-play situation continuously at moment-on-moment basis using The Observer XT 11 (Noldus). Auditory stimuli included all auditory signals that the mother provided for the child during the play session, e.g., speech and laughter. The visual signals were all the objects that the mother presented and provided for the child, e.g., showing a toy. Moreover, the analysis included whether the child looked at the mother’s activity, for example looked at the toy that the mother was showing. Tactile signals were touching, stroking, or holding the child. Descriptions for the behavioral codes for maternal sensory signals to her child are presented in the Table 2.

**TABLE 2:** Descriptions for the behavioral codes for maternal sensory signals to her child.

Sensory category	Behaviors	Description
Auditory	Maternal vocalizations	Mother makes a vocalization (“There is a ball. The ball is red.” There are two distinct vocalizations)
Tactile	Maternal touch	Touching, stroking
	Maternal holding	Holding the child
Visual	Mother manipulating objects	Mother is holding a toy or other object
	Child visually attending to maternal activities	Child is looking at a toy mother is manipulating

Inter-rater agreement at 8- and 30-months measurement points was calculated for 10% of the videotapes. Coders were blind to all other information on study participants. Inter-rater agreement for independent coders averaged 86% at 8 months and 84% at 30 months.

We quantified the extent to which sequences of maternal sensory signals were unpredictable, as described in Davis et al. (2017). Our approach focused on the conditional probabilities of transitioning between different combinations of maternal visual, auditory, and tactile sensory signals, considering all eight possible combinations of these sensory signals (presence/absence of input of each of the three types of sensory signals, see Table 3). For example, a mother might be speaking to the child while showing her a toy (auditory and visual input). If she additionally picked up the child (tactile input) so that she now provides auditory, visual, and tactile input, then this would be considered a transition to a different combination of sensory signals.

**TABLE 3:** Combinations between maternal sensory signals.

No behavior	No auditory, visual, or tactile signals
Single behavior	Only auditory
	Only visual
	Only tactile
Combination of two behaviors	Auditory and visual
	Visual and tactile
	Auditory and tactile
Combination of all three behaviors	All signals: auditory, visual, and tactile

The transitions were modeled as changes in the state of a discrete-state Markov process and the entropy rate of the process was taken as a measure of unpredictability. If one signal always followed another (e.g., touch was always followed by speech), this would be highly predictable and there would be little uncertainty for the observer (low entropy rate). If the probability of one behavior following another is random then this would be unpredictable (high entropy rate). The entropy rate ranges from 0 to 2.807, higher values indicating more unpredictable caregiving behavior. Materials regarding the calculation of the entropy rate are provided in Davis et al. (2017) and available at https://contecenter.uci.edu/shared-resources/.

The number of the transitions of maternal sensory signals was not associated with the entropy rate at 8 months (*r* = –0.08, *p* = 0.418) or 30 months (*r* = 0.063, *p* = 0.222) indicating that entropy rate is a separate construct from the quantity of transitions.

#### Maternal Sensitivity

Maternal sensitivity was assessed with Emotional Availability Scales (EA) (Biringen, 2008). EA consists of four scales regarding maternal interaction: sensitivity, structuring, non-intrusiveness and non-hostility, and two regarding the child: child responsiveness and child involvement. In this study, scale for maternal sensitivity (8 and 30 months timepoints) was used. Sensitivity coding characterized the parent’s ability to be emotionally connected with the child, to recognize the child’s interaction cues, and to respond to them appropriately and timely enough. All the scales are evaluated from 1 to 7. Scores 5.5–7 refers to healthy parent–child interaction quality, scores from 4 to 5 describes somewhat problematic such as overconnected interaction and scores from 1 to 3.5 detached or problematic interaction between parent and a child (Biringen, 2008). EA has shown significant short-term test–retest reliability (Bornstein et al., 2006b; Endendijk et al., 2019) and cross-context reliability (Bornstein et al., 2006a).

Coders were blinded to all other information about the dyad. Interrater reliability was assessed for 10% of the videotaped play episodes coded by independent coders. Intraclass correlation coefficient were 0.80 for sensitivity at 8 months and ranged from 0.83 to 0.91 at 30 months.

#### Maternal Depressive and Anxiety Symptoms

Maternal depressive symptoms were assessed with Edinburgh Postnatal Depression Scale (EPDS) (Cox et al., 1987) and maternal anxiety was assessed with the Symptom Checklist-90 (SCL-90) (Derogatis et al., 1973; Holi et al., 1998) anxiety subscale. Both measures demonstrated good internal consistency throughout the study (α = 0.82–0.89 for EPDS and 0.83–0.87 for SCL-90). Depressive symptoms were assessed repeatedly at seven time points (GW 14, 24, and 34 and 3, 6, 12, and 24 months postpartum) and anxiety symptoms at six time points (GW 14, 24, and 34 and 3, 6, and 24 months postpartum). Since we had data from 7 (EPDS) or 6 (SCL-90) time points during the pre- and postnatal periods we applied Latent Growth Mixture Modeling (LGMM; Muthén and Muthén, 2000) to inspect different trajectories of maternal depressive and anxiety symptoms. This was done to inspect the possible between-subject differences in the starting levels and rates of change in symptoms and investigate whether the courses of symptoms would differently influence maternal caregiving behavior during the pre- and postnatal periods.

The trajectories were modeled separately for depressive and anxiety symptoms with latent growth mixture modeling LGMM in Mplus (Muthén and Muthén, 2000). In this approach, growth curves of symptoms are estimated for each individual and then prototypic growth curves are identified for the whole sample. The aim is to select the latent curves, that is, “developmental patterns,” that most optimally describe the data, and effectively describe the heterogeneity in the data (as opposed to simple latent growth curve models). Also, the clinical interpretability of the latent curves is used to determine the optimal model. Individual item scores were used in the models. Participants with missing data were incorporated in the analyses with maximum likelihood under the missing-at-random assumption (Graham, 2009) in order to minimize bias (Nagin, 2005). The base data for forming the trajectories consisted of participants having interaction data at 8 and/or 30 months allowing us to retain data from *N* = 467 and *N* = 468 participants (for depressive and anxiety symptoms, respectively).

First, structural equation modeling was used to examine the factor structures of general anxiety and depressive symptom questionnaires. Model fit was evaluated by considering various descriptive goodness-of-fit indices (e.g., Marsh et al., 2004) the comparative fit index (CFI), the root mean square error of approximation (RMSEA), and the standardized root mean square residual (SRMR), which are reported with the traditional Chi-square statistics and the corresponding degrees of freedom. For the CFI, values above 0.90, represent an adequate model fit (Hu and Bentler, 1999). SRMR and RMSEA values below 0.06 and 0.08, respectively, reflect a good and acceptable fit to the data (Hu and Bentler, 1999). However, since the statistics provide various goodness-of-fit indices and the cut-off criteria for model fit evaluation are not clear-cut, researchers are recommended to simultaneously take different goodness-of-fit indices into account and treat the respective cut-off criteria as guidelines instead of golden rules (Vinni-Laakso et al., 2019).

The longitudinal Confirmatory Factor Analysis of the EPDS and SCL-90 showed adequate fit with the data [SCL-90: χ^2^(24) = 69.50, *p* > 0.05, CFI = 0.89, root mean square error of approximation = 0.04, with a slightly poor fit assessed by standardized root mean square residual (SRMR) = 0.08. The items 2 and 3, 3 and 6, and 5 and 10, and 7 and 10 were allowed to correlate to increase model fit; EPDS: χ^2^ (15) = 112.77, *p* > 0.05, CFI = 0.90, root mean square error of approximation = 0.04, standardized root mean square residual = 0.07. Consecutive Items 1 and 2, 4 and 5, and 8 and 9 were allowed to correlate to improve model fit.]. We note that the CFI value for SCL-90 was slightly below the recommended0.90. This is likely related to the fact that the follow-up period was very long (from early pregnancy to 2 years postpartum), and also the intervals between some measurement time points were long and infrequent (i.e., pregnancy weeks 12, 24, 34, and 3, 6, and 24 months postpartum for SCL-90 scores). However, we decided to accept both longitudinal CFA models as most of the indices indicated adequate or good model fit.

Second, the number of latent growth curves using LGMM models and by comparing fit indices of the models with increasing number of subgroups was studied. The following criteria were used for the decision about the optimal number of groups: Bayesian information criteria (BIC, where lower value indicates better model fit; Nylund et al., 2007), the posterior probability for each trajectory group (referring to the probability of an individual belonging to a group; here a score of >0.80 is preferred; Nagin, 2005), and entropy rate indexing classification accuracy (>0.80 indicating excellent accuracy; Lubke and Muthen, 2007). Also, theoretical, and clinical interpretability of the class solutions were used when selecting the best model.

For EPDS LGMM, the statistical indices continued to improve and/or were satisfactory up to a 3-group model (Supplementary Table 2), but the latent group sizes were larger and thus more suitable for the subsequent analyses in the 2-group model and thus it was chosen for the analyses. The two EPDS groups were labeled as “Low and stable” (*n* = 370, estimate of intercept = 2.95, estimate of slope = –0.14, *p* = 0.31), and “High and slightly decreasing” (*n* = 97, estimate of intercept = 11.24, estimate of slope = –0.41, *p* < 0.01). For SCL-90 LGMM, the 2-, 3-, and 4-group solutions were all acceptable. However, the 2-group model showed excellent fit with the data (see Supplementary Table 1) and the group sizes were sufficient for subsequent analyses, and therefore it was chosen to depict the trajectories of anxiety from early pregnancy to 2 years postpartum. We labeled the groups, based on the courses of symptoms, “Low and slightly decreasing” (*n* = 414, estimate of intercept = 2.39, estimate of slope = –0.09, *p* = 0.01) and “High and stable” (*n* = 54, estimate of intercept = 11.17, estimate of slope = 0.22, *p* = 0.44). Hence, the trajectories used in the current study analyses to describe the level and courses of psychiatric symptoms were the following for depressive symptoms “High and slightly decreasing” (mean levels for EPDS symptom scores ranged from 8.7 to 11.6)/“Low and stable” and for anxiety symptoms “High and stable” (mean levels for SCL symptom scores ranged from 9.9 to 13.7)/“Low and decreasing.”

### Data Analysis

SPSS 25 was used for the statistical analyses. Associations between background factors and maternal caregiving behavior were examined with ANOVA, *t*-test, and Pearson’s correlations. Group mean level stability between 8- and 30-months maternal unpredictability and sensitivity was analyzed with paired sample t-test and individual consistency over time with Pearson’s correlations. Relations between maternal unpredictable signals and sensitivity at 8- and 30-months were analyzed with Pearson’s correlations.

Relations between maternal depressive and anxiety symptom trajectories and maternal caregiving behavior at 30 months were analyzed with *t*-tests. General linear models were conducted to test if the associations remain when significant covariates are included for.

## Results

### The Relations Between Background Variables, Maternal Unpredictable Sensory Signals, and Sensitivity at 30 Months of Age (*n* = 356)

Higher education (*F*_2,353_ = 4.570, *p* = 0.011) and higher maternal age were related to lower unpredictability (*r* = –0.107, *p* = 0.044) at 30 months timepoint. Higher education (*F*_2,353_ = 7.465, *p* = 0.001) and higher monthly income (*F*_3,353_ = 3.060, *p* = 0.028) related to higher maternal sensitivity. Mothers were more sensitive in interactions with girls compared to boys at 30 months timepoint [*t*(354) = –2.767, *p* = 0.006]. Primiparity, gestational weeks, birth weight and Apgar scores were unrelated to maternal unpredictable signals and sensitivity at 30 months of child’s age.

### Stability of Maternal Unpredictable Sensory Signals and Sensitivity Between Infancy (8 Months) and Toddlerhood (30 Months) (*n* = 103)

Mothers were less unpredictable at 30 months compared to 8 months measurement point [*t*(102) = 4.737, *p* < 0.001]. In contrast, there was no mean level change in maternal sensitivity between 8 and 30 months timepoints. See Table 4.

**TABLE 4:** Group comparisons for maternal unpredictable signals and sensitivity at 8 and 30 months (*n* = 103).

	8-Month mother-child interaction	30-Month mother-child interaction	

	*M* (*SD*)	*M* (*SD*)	*t* (df)
Unpredictable sensory signals (entropy rate)	0.87 (0.16)	0.79 (0.13)	4.737 (102), *p* < 0.001
Sensitivity	5.36 (1.32)	5.27 (1.05)	0.658 (102), *p* = 0.512

Pearson correlations showed that unpredictability (*r* = 0.296, *p* = 0.002) and maternal sensitivity (*r* = 0.293, *p* = 0.003) was moderately consistent between the 8 and 30 months timepoints.

### Relations Between Unpredictability of Maternal Sensory Signals and Sensitivity in Infancy (8 Months, *n* = 103) and Toddlerhood (30 Months *n* = 356)

Higher maternal unpredictability associated with lower maternal sensitivity at both 8 (*r* = –0.279, *p* = 0.004) and 30 months timepoints (*r* = –0.146, *p* = 0.006).

### Maternal Depressive and Anxiety Symptoms Trajectories and Maternal Unpredictable Sensory Signals and Sensitivity at 30 Months (*n* = 356)

The trajectory classes of depressive or anxiety symptoms were not related to maternal unpredictable sensory signals at 30 months timepoint. However, the class of “high and slightly decreasing” depressive symptoms throughout pre- and postnatal periods (*n* = 68) was associated with lower maternal sensitivity *M*(*SD*) = 4.66 (1.24), [*t*(354) = –4.132, *p* < 0.001] at 30 months timepoint compared to the class of “low and stable” depressive symptoms (*n* = 288) *M(SD)* 5.30(1.11). Similarly, the class “high and stable” anxiety symptoms throughout pre- and postnatal periods (*n* = 33) related to lower maternal sensitivity at 30 months timepoint M(SD) = 4.79(1.32), [*t*(354) = –2.013, *p* = 0.045] compared to the class “low and decreasing” anxiety symptoms (*n* = 323) *M(SD)* 5.21(1.14).

General linear models were conducted to see whether elevated depressive or anxiety symptoms predict maternal sensitivity at 30 months timepoint when relevant covariates are included for (i.e., education, income, infant sex). Elevated depressive symptoms associated significantly with lower maternal sensitivity (β = –0.603, *p* < 0.001) even after inclusion of covariates. In this model, higher education (low vs. high β = –0.488, *p* = 0.002; middle vs. high β = –0.306, *p* = 0.032) related to higher sensitivity and infant sex (boy) (β = –0.288, *p* < 0.016) to lower sensitivity, see Table 5. Model explained 9.7% of the variance in sensitivity. Similarly, elevated anxiety symptoms associated with lower maternal sensitivity (β = –0.416, *p* = 0.046) when covariates were included. Higher education (low vs. high β = –0.419, *p* = 0.002; middle vs. high β = –0.326, *p* = –0.024) associated with higher sensitivity and infant sex (boy) (β = –0.318, *p* = 0.009) associated with lower sensitivity, see Table 6. Model explained 6.5% of the variance in sensitivity.

**TABLE 5:** Maternal sensitivity 30 months and depressive symptoms (EPDS).

	β	*p*	95% CI	$\eta_{\text{p}}^{2}$
**Education**				
Low	–0.488	0.002	–0.795, –0.182	0.027
Middle	–0.306	0.032	–0.585, –0.027	0.013
High	0^a^			
**Income**				
1500 or less	–0.194	0.679	–1.114, 0.726	0.000
1501–2500	0.103	0.824	–0.805, 1.010	0.000
2501–3500	–0.212	0.661	–1.161, 0.738	0.001
>3500	0^a^			
Infant sex (boy)	–0.288	0.016	–0.522, –0.054	0.017
**EPDS**				
High	–0.603	<0.001	–0.897, –0.309	0.045
Low	0^a^			

*^a^Reference group.*

**TABLE 6:** Maternal sensitivity 30 months and anxiety symptoms (SCL).

	β	p	95% CI	$\eta_{\text{p}}^{2}$
**Education**				
Low	–0.419	0.002	–0.803, –0.179	0.027
Middle	–0.326	0.024	–0.610, –0.042	0.014
High	0^a^			
**Income**				
1500 or less	–0.161	0.736	–1.098,0.776	0.000
1501–2500	0.128	0.785	–0.796, 1.052	0.000
2501–3500	–0.188	0.702	–1.154, 0.778	0.000
>3500	0^a^			
Infant sex (boy)	–0.318	0.009	–0.556, –0.079	0.019
**SCL**				
High	–0.416	0.046	–0.824, –0.007	0.011
Low	0^a^			

*^a^Reference group.*

## Discussion

The current study examined the development and relations of a novel aspect of caregiving, unpredictable maternal sensory signals (Davis et al., 2017; Vegetabile et al., 2019) and a more traditional aspect of maternal sensitivity (Biringen, 2008; Biringen et al., 2014) from infancy to toddlerhood. Moreover, it was studied how different courses of maternal pre- and postnatal depressive and anxiety symptoms predict the quality of maternal caregiving behaviors in toddlerhood. Previous research has focused mainly on emotional aspect of caregiving behavior such as sensitivity with fewer studies evaluating the patterns of maternal behaviors or changes in these caregiving aspects across infancy and childhood. It was found that, on average, maternal unpredictability decreased from infancy to toddlerhood, whereas a mean stability on maternal sensitivity was observed. Moreover, both maternal unpredictability and sensitivity were moderately associated between 8 and 30 months suggesting some consistency within an individual. Further, unpredictability and sensitivity were modestly to moderately associated with one another throughout infancy and toddlerhood.

Furthermore, courses of depressive and anxiety symptoms from pregnancy to the postnatal period were not related to maternal unpredictability in toddlerhood. However, maternal elevated depressive and anxiety symptoms (vs. low and stable symptoms) modeled longitudinally throughout pre- and postnatal periods were related to lower maternal sensitivity in interaction with their toddler when significant covariates (education, income, infant sex) were included for.

Interestingly, maternal unpredictability decreased on average from infancy to toddlerhood phase. There are no previous studies investigating the normative development of the unpredictability of maternal sensory signals throughout child development. However, our finding supports the possibility that the quality of maternal caregiving behavior may improve during child development on average (Biringen et al., 1999; Kemppinen et al., 2006) as mother adapts to motherhood and learns parenting skills when the child develops. Moreover, the change in maternal unpredictability on average could be related also to child developmental skills such as language, self-regulation, and executive functions, which might increase the fluent and coherent parenting behaviors. The finding that mean level of maternal sensitivity did not significantly change between child’s infancy and toddlerhood is consistent with previous results showing that on average maternal sensitivity does not change with child age (Célia et al., 2018) across the first years of child’s life. In contrast, our results did not support the view that maternal caregiving quality may decrease on average along with parenting challenges caused by toddlerhood behavioral development (Bornstein et al., 2011).

At individual level, as hypothesized, maternal unpredictability and sensitivity showed moderate level of stability (Dallaire and Weinraub, 2005; Kemppinen et al., 2006; Bornstein et al., 2011; Hall et al., 2015). Our results suggest that mothers who are unpredictable or sensitive in the interaction with their infant are likely to be also unpredictable or sensitive in interaction with their toddler. These results replicate the previous findings that quality of maternal care, once established in infancy, is a relatively stable maternal characteristic across child development, even though the child matures. To our knowledge, this is also the first study to show that unpredictability of maternal care followed this same pathway of individual stability as maternal sensitivity. However, more studies and replications are needed to understand the normative development of unpredictability of maternal care across child development. Analyses comparing two current study paradigms also suggested that unpredictability of maternal signals and sensitivity are from modestly to moderately interrelated across infancy and toddlerhood, but they are clearly separate aspects of parenting, as hypothesized (Davis et al., 2017).

However, beyond the mean-level changes and individual stability, it remains of interest which factors explain the individual differences in these aspects of parenting, specifically the unpredictability of care. High maternal depressive or anxiety symptoms during pregnancy and child early years were not related to the unpredictability of maternal signals in toddlerhood. This is in contrast the prior findings regarding unpredictability of care (Davis et al., 2017, 2019). However, in previous studies only concurrent symptoms were measured rather than courses of symptoms from pregnancy to postpartum. It may be that unpredictable care is a more multifactorial system and several maternal risk factors, e.g., low self-regulation capacity and anxiety symptoms should be considered when studying the underlying maternal factors (Holmberg et al., 2020). However, based on the current study results it may be suggested that unpredictability of maternal signals is not as sensitive to maternal mood (high vs. low) as maternal sensitivity.

Our finding that elevated depressive and anxiety symptoms throughout pre- and postnatal period related to lower maternal sensitivity in toddlerhood adds to the growing evidence that chronic depressive or anxiety symptoms starting already during pregnancy (or before) may have effects for the quality of maternal care until child early years (Easterbrooks et al., 2000; Lovejoy et al., 2000; Parfitt et al., 2013; Field, 2017). Maternal prenatal distress may negatively affect psychological transition to motherhood via straight biological effects on the expectant mother’s brain functions (Brunton and Russell, 2008; Hillerer et al., 2014) and disturbing the natural development of maternal-fetal attachment (Dayton et al., 2010; Ahlqvist-Björkroth et al., 2016). Moreover, prenatal distress may affect fetal development via prenatal programming, which may compromise the quality of postnatal mother–infant interaction (Sandman et al., 2011). In addition, the results are in line with recent studies pointing out the importance to focus also on anxiety, in addition to depressive symptoms, when studying the quality of caregiving behavior (Easterbrooks et al., 2000; Lovejoy et al., 2000; Stein et al., 2012; Tietz et al., 2014).

Although not the main focus of this study, it was found that higher maternal education related to lower unpredictability and higher maternal sensitivity and higher maternal age to lower unpredictability in toddlerhood. These results are in line with previous research showing that low socioeconomic situation may be a risk factor for beneficial caregiving behaviors (Conger et al., 2011; Roubinov and Boyce, 2017). Moreover, mothers were more sensitive in interactions with the girls compared to boys during toddlerhood. The effects of child gender for caregiving quality are sparsely studied, but there are some evidence that especially mother-girl dyads perform most harmoniously (Lovas, 2005; Bornstein et al., 2011), which has been related to earlier maturation of the girls and socializing norms possibly favoring girls vs. boys.

The current study has several strengths. Mother-child interaction was assessed longitudinally in a relatively large sample using two different observational measurements with high inter-rater agreements. Moreover, maternal depressive and anxiety symptoms were assessed in a longitudinal follow-up throughout pre- and postnatal period. However, there are some limitations to be considered in this study. Participants in this study had higher education, high monthly income and higher maternal age compared to non-participating mothers, which limits generalizability. Despite of our relatively large sample size for depressive and anxiety symptoms, the latent growth mixture modeling was not able to detect large enough samples to study subtle differences in the trajectories of symptoms. Also, some fit indices in the longitudinal CFA of the symptom scores were slightly below adequate whereas most of them showed adequate or good fit. This was likely related to our long follow-up period with infrequent assessment points toward the end of the follow-up. However, we see merit in this longitudinal modeling strategy taking use of maximal amount of data. Moreover, we were not able to use multidimensional statistical modeling to explore individual trajectories of maternal caregiving behavior from infancy to toddlerhood due to the small sample and only two measurement points. Furthermore, three assessment points of mother-infant interaction (also including assessment from early months) would offer more complete picture of the development of mother–infant interaction.

The results show that both unpredictability of maternal patterns of sensory signals and maternal sensitivity are moderately stable traits from infancy onward, which gives a strong rationale to start interventions focusing on mother-child relationship during infancy (Biringen et al., 2014) or already during pregnancy (Salo et al., 2019). In addition, chronically elevated maternal symptoms of distress starting during pregnancy seems to constitute a risk factor for the maternal sensitivity at least until the child toddlerhood years. Moreover, the first 1000 days are crucial for child neurodevelopment (Derbyshire and Obeid, 2020) and the early environment and the quality of maternal care plays an important role in this development. Current results strengthen the view that screening both depressive and anxiety symptoms during pregnancy period and supporting mothers should be of special focus in clinical settings.

## Data Availability Statement

The datasets presented in this article are not readily available because there are strict legal rules in terms of data sharing in the medical faculty at the University of Turku. The dataset is available upon request. Requests to access the datasets should be directed to Juho Pelto, jepelt@utu.fi.

## Ethics Statement

The studies involving human participants were reviewed and approved by the Ethics Committee of the Hospital District of Southwest Finland. Written informed consent to participate in this study was provided by the participants’ legal guardian/next of kin.

## Author Contributions

EH: writing – original draft. E-LK: investigation and writing – review and editing. EPD, MP, and RK: supervision and writing – review and editing. SN: investigation, writing – review and editing, and methodology. HH: investigation and writing – review and editing. LK and HK: resources, funding acquisition, conceptualization, and writing – review and editing. All authors contributed to the article and approved the submitted version.

## Conflict of Interest

The authors declare that the research was conducted in the absence of any commercial or financial relationships that could be construed as a potential conflict of interest.

## Publisher’s Note

All claims expressed in this article are solely those of the authors and do not necessarily represent those of their affiliated organizations, or those of the publisher, the editors and the reviewers. Any product that may be evaluated in this article, or claim that may be made by its manufacturer, is not guaranteed or endorsed by the publisher.

## Footnotes

1
www.thl.fi
