# Supplementary material for: The Connection and Development of Unpredictability and Sensitivity in Maternal Care Across Early Childhood
Source: Front Psychol. 2022 Mar 7;13:803047. doi: 10.3389/fpsyg.2022.803047 (PMC8940198; doi:10.3389/fpsyg.2022.803047)
Supplement: Supplementary file 1 [file Table_1.DOCX]

| Table S2. Model Fit Indices for EPDS classes | | | | | | |
| --- | --- | --- | --- | --- | --- | --- |
|  | Log L. | AIC | BIC | Entropy | Class Proportions | Average Latent Class Posterior Probabilities |
| EPDS |  |  |  |  |  |  |
| 1 Class | -7303.148 | 14630.296 | 14680.052 | 1.00 | 1.000 | 1.000 |
| 2 Class | -7241.460 | 14512.919 | 14575.114 | .86 | .21/.79 | .92/.97 |
| 3 Class | -7213.416 | 14462.833 | 14537.467 | .91 | .24/.01/.75 | .92/.76/.76 |
| 4 Class | -7199.543 | 14441.086 | 14528.159 | .86 | .13/.67/.18/.06 | .89/.95/.83/1 |

| Table S1. Model Fit Indices for SCL classes | | | | | | |
| --- | --- | --- | --- | --- | --- | --- |
|  | Log L. | AIC | BIC | Entropy | Class Proportions | Average Latent Class Posterior Probabilities |
| SCL |  |  |  |  |  |  |
| 1 Class | -6308.357 | 12638.713 | 12684.346 | 1.000 | 1.000 | 1.000 |
| 2 Class | -6197.959 | 12423.917 | 12481.996 | .95 | 12/.88 | .96/.99 |
| 3 Class | -6128.082 | 12290.164 | 12360.688 | .95 | .83/.07/.10 | .99/.95/.91 |
| 4 Class | -6074.751 | 12189.502 | 12272.472 | .96 | .07/.01/.80/.12 | .94/1/.99/.92 |
